# Hydroxyapatite Scaffold and Bioactive Factor Combination as a Tool to Improve Osteogenesis, In Vitro and In Vivo Experiments Using Phage Display Technology

**DOI:** 10.3390/ijms26157040

**Published:** 2025-07-22

**Authors:** Debora Lo Furno, Ivana R. Romano, Vincenzo Russo, Maria Giovanna Rizzo, Giuliana Mannino, Giovanna Calabrese, Rosario Giuffrida, Simona D’Aprile, Lucia Salvatorelli, Gaetano Magro, Riccardo Bendoni, Laura Dolcini, Agata Zappalà, Salvatore P. P. Guglielmino, Sabrina Conoci, Rosalba Parenti

**Affiliations:** 1 Department of Biomedical and Biotechnological Sciences (BIOMETEC), University of Catania, Via Santa Sofia 97, 95123 Catania, Italy; lofurno@unict.it (D.L.F.); ivanarobertaromano@yahoo.it (I.R.R.); vincenzo.russo@unict.it (V.R.); giuffros@unict.it (R.G.); simonettadap@gmail.com (S.D.); azappala@unict.it (A.Z.); parenti@unict.it (R.P.); 2 Department of Chemical, Biological, Pharmaceutical and Environmental Sciences (ChiBioFarAm), University of Messina, Viale F. Stagno d’Alcontres 31, 98166 Messina, Italy; mariagiovanna.rizzo@unime.it (M.G.R.); giovanna.calabrese@unime.it (G.C.); sguglielm@unime.it (S.P.P.G.); 3 Department of Medicine and Surgery, University of Enna “Kore”, 94100 Enna, Italy; giuliana.mannino@unikore.it; 4 Department of Medical, Surgical Sciences and Advanced Technologies “G.F. Ingrassia”, Anatomic Pathology, University of Catania, Via Santa Sofia 87, 95123 Catania, Italy; lucia.salvatorelli@unict.it (L.S.); g.magro@unict.it (G.M.); 5 Fin-Ceramica Faenza SPA, Via Granarolo 177/3, 48018 Faenza, Italy; riccardo.bendoni@finceramica.it (R.B.); ldolcini@finceramica.it (L.D.); 6 Department of Chemistry “Giacomo Ciamician”, University of Bologna, Via Selmi 2, 40126 Bologna, Italy

**Keywords:** hydroxyapatite scaffolds, human adipose-derived stem cells, osteogenic differentiation, phage display, female BALB/c mice, regenerative medicine

## Abstract

Mesenchymal stem cells have been widely investigated in the field of regenerative medicine and also used as a model to study the differentiation-induction properties of a variety of biomaterials. This study evaluates the osteoinductive potential of novel hydroxyapatite scaffolds functionalized with a phage-displayed peptide (SC1) selected via biopanning for its similarity to bone matrix proteins. The peptide, identified through sequence alignment as a mimotope of osteonectin (SPARC), was used to functionalize scaffolds. Results from SC1 were gathered at different time points (14, 28 and 46 days) and compared with those from nonfunctionalized hydroxyapatite (HA) scaffolds. In vitro experiments, by seeding human adipose-derived stem cells (hASCs), indicated satisfactory biocompatibility for both types of scaffolds. Histochemical observations showed that SC1, better than HA scaffolds, was able to improve hASC osteogenic differentiation, as evaluated through Alizarin Red staining (showing on average a darker staining of 100%). An increase was also observed, especially at early stages (14 days), for osterix (up to 60% increase) and osteonectin immunoexpression (up to 50% increase). In in vivo experiments, cell-free scaffolds of both types were subcutaneously implanted into the backs of mice and analyzed after 2, 4, 8 and 16 weeks. Also, in this case, SC1 more effectively promoted the osteogenic differentiation of infiltrated resident cells. In particular, increased immunoexpression of osterix and osteonectin (+30% and 35%, respectively) was found already at 2 weeks. It can be concluded that SC1 scaffolds may represent a valuable tool to address critical-sized bone defects.

## 1. Introduction

In physiological conditions, bone tissue possesses remarkable self-repair capacity. However, nonunion or malunion fractures can sometimes occur, bone defects can be wider than 2 cm or the loss of bone circumference exceeds 50% [1,2]. In these cases, autologous grafting is the gold standard as it does not raise histocompatibility and immunogenicity issues and also features osteoinductive, osteogenic and osteoconductive properties [1]. Unfortunately, the harvesting of autologous bone often causes secondary damage to the donor site and surgical-procedure-related risks. Moreover, when the defect is larger than the available harvestable bone, autologous transplantation is not practicable. In this context, regenerative medicine offers several advantages both through biomaterials and adult mesenchymal stem cells (MSCs) [3,4]. Moreover, MSCs can be used to study various scaffold-related physiological properties such as cell growth, migration and differentiation [5,6].

MSCs are multipotent stem cells present in specific zones called niches [7], where they guarantee tissue homeostasis. They can easily differentiate into cells of mesodermal origin or to other cell types such as neural or epithelial cells [8]. First isolated from the bone marrow [9], MSCs are present in many other tissues [10]. Numerous studies have focused on adipose-derived mesenchymal stem cells (ASCs) because they can be easily harvested with negligible pain for patients [11,12,13].

In the field of bone defect treatments, a combination of scaffolds, cells, molecules and different factors are being developed to support satisfactory bone formation. Particularly interesting is the development of biomaterials that promote bone regeneration in situ, avoiding donor site morbidity [14,15]. Collagen-based scaffolds are employed because they provide certain advantages. They offer mechanical support during cell proliferation and differentiation into osteo/chondral cells, as well as the production of cartilaginous matrix that requires a three-dimensional environment.

Hydroxyapatite (HA, Ca_10_(OH)_2_(PO_4_)_6_)-based scaffolds have been tested because they are particularly suitable, showing a structure similar to the bone. HA represents the main component of the mineral phase, binds the organic matrix and is a nontoxic material [5]. Thus, these biodegradable scaffolds mimic the physiological microenvironment and provide a 3D support able to host cells, also stimulating their proliferation and differentiation, both in vitro and in vivo [16]. HA scaffolds can be supplemented with molecules that enhance their osteoconductivity and osteoinductivity. Lately, research has been focused on phage peptides mimicking bone matrix proteins. Phage display (PD) technology is an innovative approach in which functional exogenous peptides are exposed on the capsid surface of a bacteriophage as a fusion product with endogenous proteins of the phage coat. In particular, several groups used PD technology to select tissue-specific peptides able to stimulate cell adhesion, proliferation and differentiation and improve osteo-chondral regeneration, by identifying regulatory proteins that modulate osteoblast differentiation (TGFβ receptor-interacting protein 1; TRIP-1), by using a PD library [17,18,19].

These peptides have demonstrated advantages such as high biocompatibility, the ability to be immobilized directly on chondro- and osteo-inductive nanomaterials, improving cell attachment, differentiation, development, and finally, the regeneration of osteochondral tissue [20,21].

In the present study, we used phage-display technology to identify a peptide mimicking osteonectin, also known as secreted protein acidic and rich in cysteine (SPARC). We used this matrix protein involved in osteogenesis to functionalize HA scaffolds. The functional properties of these peptide-coated scaffolds (SC1) were compared to non-functionalized HA scaffolds, both in vitro and in vivo.

For in vitro experiments, the biocompatibility and the osteoinductive properties of the two types of scaffolds were tested using human ASCs, chosen for their propension to osteogenic differentiation when cultured in a specific osteoinductive medium. In in vivo experiments, the same properties were investigated after the subcutaneous implantation of both cell-free scaffolds into the backs of mice.

## 2. Results

### 2.1. Peptide Alignment Characteristics

**Figure 1:**
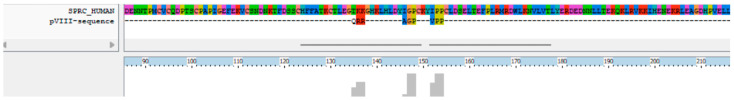
Alignment of the selected peptide with human SPARC.

Among the clones sequenced after the third round, the one expressing the peptide QRRAGPVPP was selected for scaffold functionalization. The amino acid sequence of this peptide was aligned to the human SPARC protein using ClustalX 2.1 and other alignment tools. The analysis revealed sequence homology with the C-terminal region of SPARC (Figure 1).

Specifically, matching residues included the motifs QRR, AGP and VPP, which aligned with conserved regions of the SPARC sequence. The alignment was supported by localized conservation scores, with the corresponding segments in SPARC spanning positions 135 to 155.

### 2.2. Scaffold Biocompatibility In Vitro

**Figure 2:**
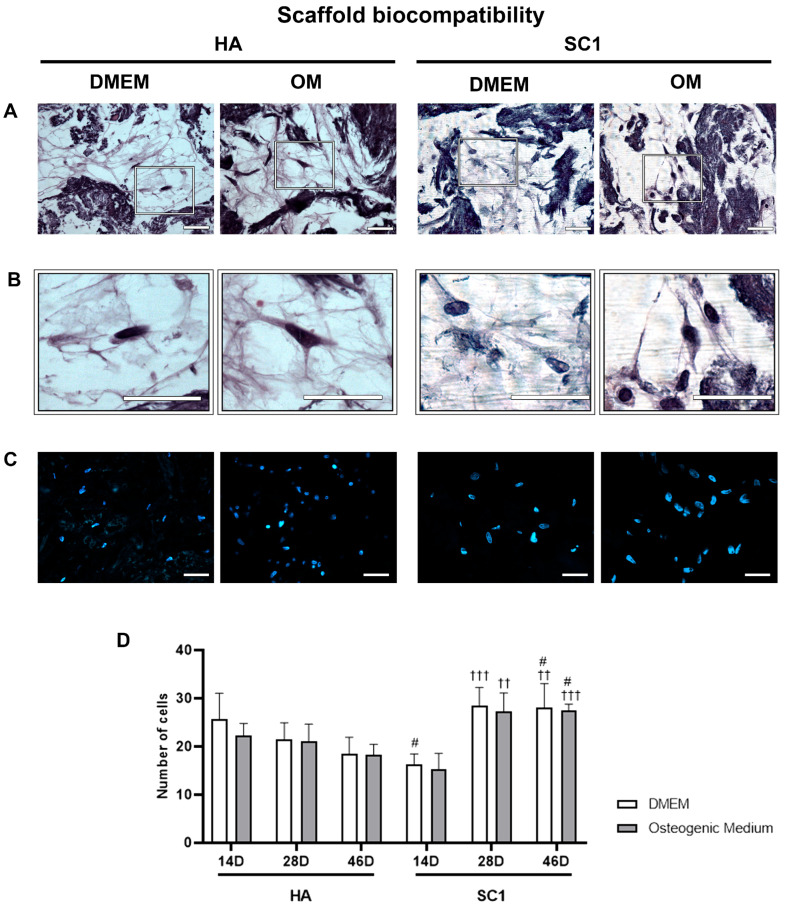
hASC viability inside HA and SC1 scaffolds. Photomicrographs show hematoxylin/eosin (**A**,**B**) and DAPI (**C**) staining in scaffold sections at 46 days from seeding. The presence of cells inside the scaffolds is better shown in the enlargements (**B**). Quantitative estimations of cell number in the two types of scaffolds and in the different culture conditions is reported in the histograms (**D**), at each detection time (14, 28 and 46 days). Scale bars: 50 µm. No significant differences were found comparing OM vs. DMEM for the same type of scaffold at the same time points. Significant differences were instead observed when comparing SC1 vs. HA: # SC1 vs. HA, same treatment at the same time point (# *p* < 0.05); † vs. 14 days, for the same scaffold and the same treatment (†† *p* < 0.01, ††† *p* < 0.005). For each experimental condition, values are reported as the mean ± SD. Statistical analysis was performed via three-way ANOVA (*n* = 18). Abbreviations: hASC = human adipose-derived mesenchymal stem cell; HA = hydroxyapatite scaffold; SC1 = scaffold functionalized with phage clones; DMEM = Dulbecco’s modified Eagle’s medium; OM = osteogenic medium.

Scaffold biocompatibility was assessed based on the ability of hASCs to adhere and invade the bone-like single-layer SC1 and HA scaffolds. Data were gathered at 14, 28 and 46 days after cell seeding from three independent experiments, each performed in triplicate. Figure 2 shows results obtained after hematoxylin/eosin (A) and DAPI (B) staining. It was found that hASCs were able to infiltrate into and survive in the scaffolds in both experimental culture conditions: Dulbecco’s modified Eagle’s medium (DMEM) or osteogenic medium (OM). The enlargements below the microphotographs more clearly show cells inside the scaffolds. Nuclei counts (C) revealed that the number of cells invading the scaffolds was not significantly different when comparing DMEM vs. OM for the same type of scaffold at the same time points, whereas differences were observed between SC1 vs. HA. Increased cell populations were found when cells were grown in SC1, comparing cell counts at 28 and 46 days to those at 14 days.

### 2.3. Scaffold Osteo-Inductive Effects on hASCs In Vitro

**Figure 3:**
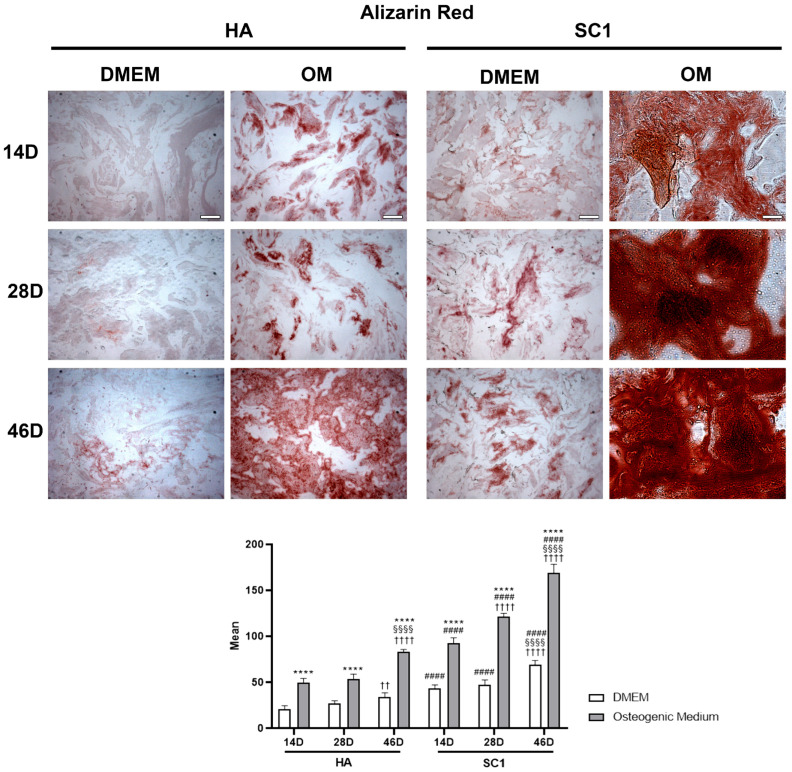
Photomicrographs showing Alizarin Red S staining of HA and SC1 scaffolds. Results were collected at different time points (14, 28 and 46 days) after hASC seeding, maintained in basal DMEM or in osteogenic medium. Both the images and quantitative measurements reported in the histograms below show that, even in basal conditions, a significant increased density is detectable when hASCs were seeded in SC1, in a time-dependent manner. As expected, OM treatment induced a stronger staining intensity, both in HA and, even more, in SC1. Scale bars: 50 µm. * OM vs. DMEM for the same type of scaffold at the corresponding time point (**** *p* < 0.001); # SC1 vs. HA, for the same treatment at the same time point (#### *p* < 0.001); † vs. 14 days, for the same scaffold and the same treatment (†† *p* < 0.01, †††† *p* < 0.001); § vs. 28 days, for the same scaffold and the same treatment (§§§§ *p* < 0.001). For each experimental condition, values are reported as the mean ± SD. Statistical analysis was performed via three-way ANOVA (*n* = 18). Abbreviations: hASC = human adipose-derived mesenchymal stem cell; HA = hydroxyapatite scaffold; SC1 = scaffold functionalized with phage clones; DMEM = Dulbecco’s modified Eagle’s medium; OM = osteogenic medium.

The osteo-inductive ability of the two types of scaffolds (HA and SC1) was evaluated by monitoring osteoblast differentiation of hASCs seeded in the scaffolds. For this purpose, Alizarin Red S staining (which reveals the extracellular matrix calcium) [22,23] and the immunoexpression of osterix and osteonectin (typical osteoblast markers) were tested. Data were gathered from three independent experiments, each performed in triplicate. Although to different extents, Alizarin Red S staining gradually increased in a time-dependent manner under all conditions (Figure 3), both in DMEM and in OM, at 14, 28 and 46 days after cell seeding. In HA scaffolds, when hASCs were cultured in DMEM, the weak staining present at day 14 was increasingly pronounced at day 28 and 46. As was expected, more intense staining was detected at each time point with OM. In SC1 scaffolds, significantly darker staining was observed in both culture conditions and at the different time points. The histograms in Figure 3 show an average increase of 100% at any time point with either DMEM or OM. In particular, the mineralization process visible in DMEM indicates that SC1 is able per se to stimulate hASC osteogenic differentiation; this process was strongly enhanced with OM.

**Figure 4:**
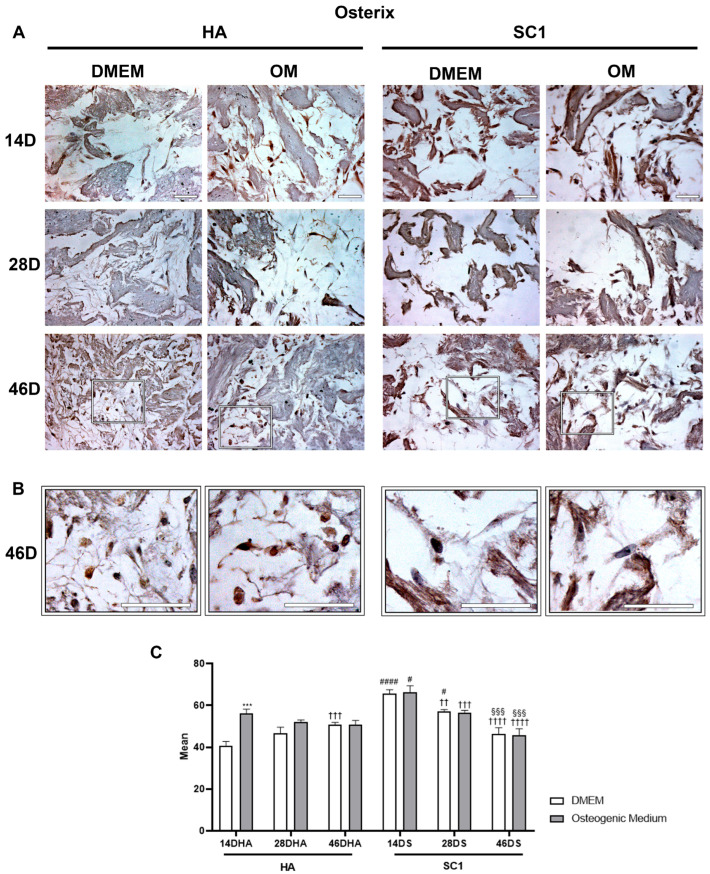
Photomicrographs showing osterix immunostaining of HA and SC1 scaffolds. Results were gathered at different time points (14, 28 and 46 days) after hASC seeding, either in basal DMEM or in osteogenic medium (**A**). Some enlargements at 46 days of culture for each condition are reported in panel (**B**). Both the images and the quantitative measurements (histograms in (**C**)) show that in basal conditions, a significant increase in expression is detectable when hASCs were seeded in SC1. While osterix expression in HA increased over time (28 and 46 days), an opposite trend could be observed for SC1. OM treatment induced stronger osterix expression only in HA scaffolds. Scale bars: 50 µm. * OM vs. DMEM, for the same type of scaffold at the same time point (*** *p* < 0.005); # SC1 vs. HA, for the same treatment at the same time point (# *p* < 0.05, #### *p* < 0.001); † vs. 14 days, for the same scaffold and the same treatment (†† *p* < 0.01, ††† *p* < 0.005, †††† *p* < 0.001); § vs. 28 days, for the same scaffold and the same treatment (§§§ *p* < 0.005). For each experimental condition, values are reported as the mean ± SD. Statistical analysis was performed via three-way ANOVA (*n* = 18). Abbreviations: hASC = human adipose-derived mesenchymal stem cell; HA = hydroxyapatite scaffold; SC1 = scaffold functionalized with phage clones; DMEM = Dulbecco’s modified Eagle’s medium; OM = osteogenic medium.

Immunohistochemistry for ASC osterix and osteonectin expression indicated that these osteogenic markers were variously modulated in the different conditions. As illustrated in Figure 4, osterix immunoexpression at 14 days after seeding clearly shows that, even in DMEM, significantly increased expression was found in hASCs cultured in SC1 as compared to HA scaffolds (up to 60% increase). At longer timepoints (28 and 46 days), a different trend was observed: while osterix expression gradually increased in HA, the opposite trend was observed for SC1. This is not surprising, considering that osterix is an early marker of pre-osteoblasts. It can be assumed that a slower osteo-induction occurs when ASCs are seeded in HA, still increasing from 14 to 46 days. Instead, a faster differentiation process induced by SC1 reaches an earlier peak at 14 days, diminishing at later timepoints (28 and 46 days), when cells enter a more mature stage. Results obtained using OM corroborate this interpretation. In fact, the increased osterix levels in HA at 14 days indicate a faster differentiation process, showing a decrease in the following days. On the other hand, OM treatment in SC1 did not show significant differences compared to osterix expression in DMEM conditions. Probably, the high degree of differentiation induced by SC1 alone at day 14 could not be further enhanced.

**Figure 5:**
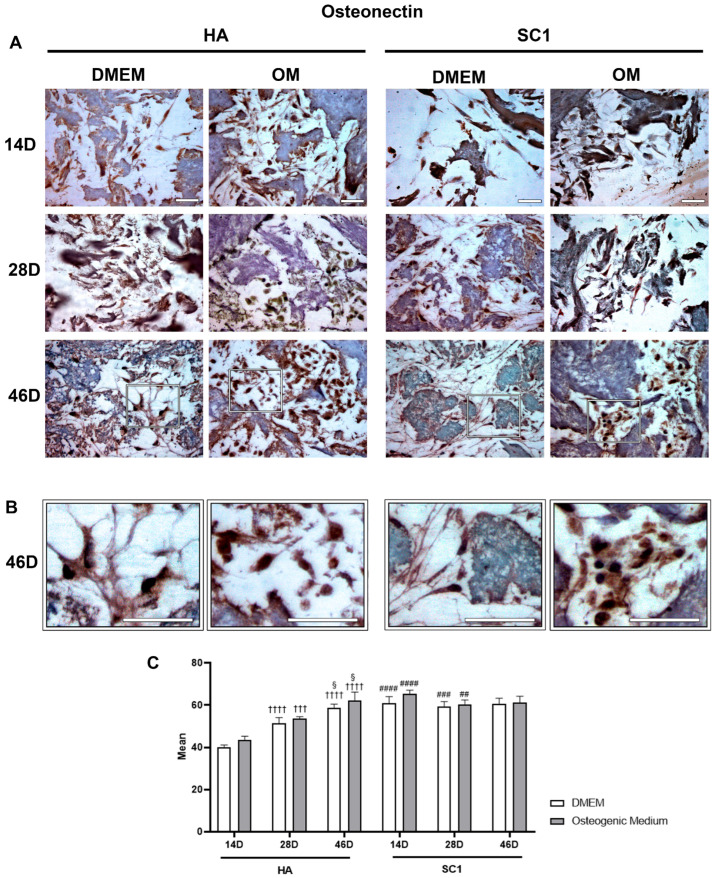
Photomicrographs showing osteonectin immunostaining of HA and SC1 scaffolds. Results were gathered at the different time points (14, 28 and 46 days) after hASC seeding, either in basal DMEM or in osteogenic medium (**A**). Some enlargements at 46 day of culture for each condition are reported in panel (**B**). Both the images and the quantitative measurements (histograms in (**C**)) show that in basal conditions, a significant increase in expression is detectable when hASCs were seeded in SC1. In particular, osteonectin expression in HA increased over time (28 and 46 days), either when hASCs were cultured in DMEM or OM. A different trend could be observed for SC1. The high marker expression present at 14 days is largely maintained at longer detection times. Scale bars: 50 µm. No significance was found comparing OM vs. DMEM for the same type of scaffold at the same time points. Significant differences were instead observed when comparing SC1 vs. HA: # SC1 vs. HA, for the same treatment at the same time point (## *p* < 0.01, ### *p* < 0.005, #### *p* < 0.001); † vs. 14 days, for the same scaffold and the same treatment (††† *p* < 0.005, †††† *p* < 0.001); § vs. 28 days, for the same scaffold and the same treatment (§ *p* < 0.05). For each experimental condition, values are reported as the mean ± SD. Statistical analysis was performed via three-way ANOVA (*n* = 18). Abbreviations: hASC = human adipose-derived mesenchymal stem cell; HA = hydroxyapatite scaffold; SC1 = scaffold functionalized with phage clones; DMEM = Dulbecco’s modified Eagle’s medium; OM = osteogenic medium.

Osteonectin immunoexpression is illustrated in Figure 5. Observations carried out at 14 days after ASC seeding clearly show that osteonectin levels detected in DMEM cultures were also significantly higher in SC1 compared to HA scaffolds (up to 50% increase). In HA, marker expression increased over time, with more evident effects at 46 days, particularly when OM was used. In SC1, a different trend was observed: the high marker expression present at 14 days is largely maintained at longer detection time. This trend was observed for both DMEM and OM cultures. These results confirm that, compared to HA, SC1 accelerates osteogenic differentiation.

### 2.4. Osteogenic Differentiation In Vivo

**Figure 6:**
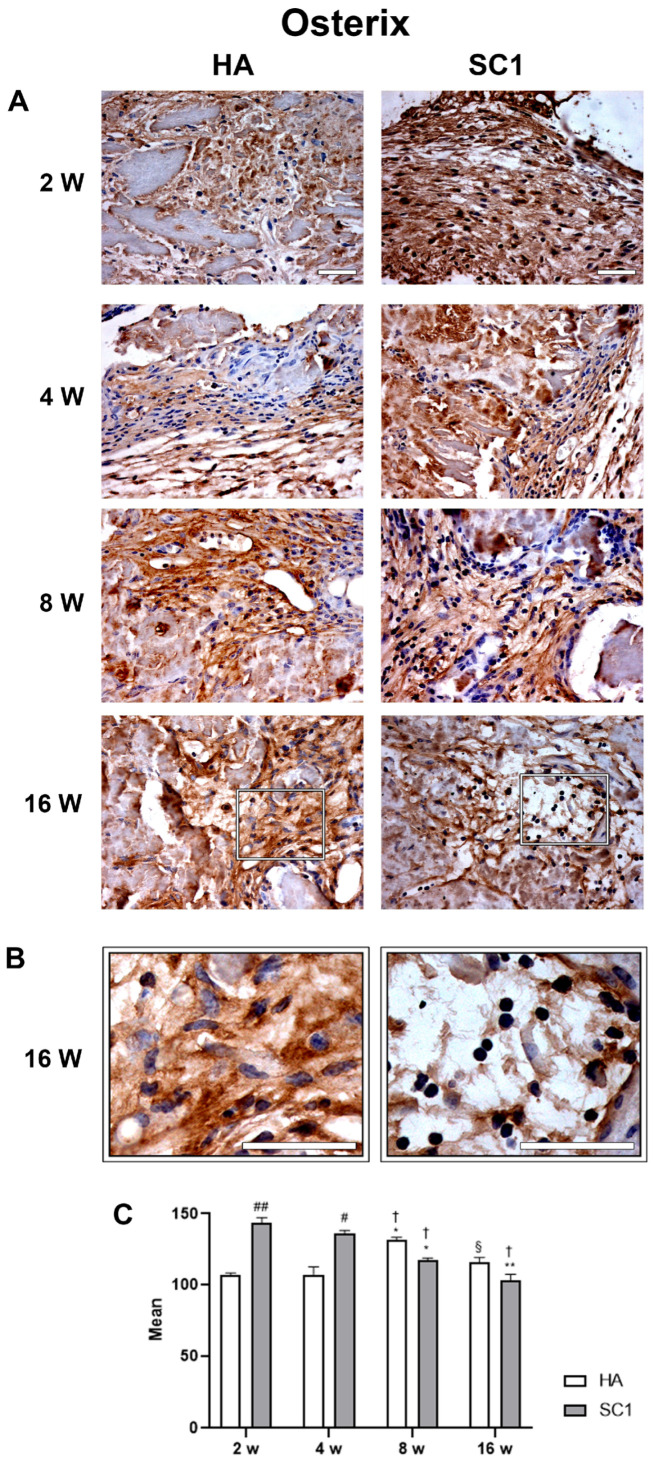
Photomicrographs showing osterix immunostaining of HA and SC1 scaffolds, at different time points (2, 4, 8 and 16 weeks) after subcutaneous implantation into the backs of mice (**A**). Some enlargements at 16 weeks after implantation of each scaffold are reported in panel (**B**). Both the images and the quantitative measurements (histograms in (**C**) show that osterix immunoreactivity detected at 2 weeks in HA, progressively increased up to 8 weeks, showing a decline at the last detection time (16 weeks). In SC1, osterix immunoreactivity at 2 weeks was significantly higher than HA, but showing a decrease at longer detection times. Scale bars: 50 µm. # SC1 vs. HA at the same time point (# *p* < 0.05, ## *p* < 0.01); * vs. 2 weeks for the same type of scaffold (* *p* < 0.05, ** *p* < 0.01); † vs. 4 weeks for the same type of scaffold († *p* < 0.05); § vs. 8 weeks for the same type of scaffold (§ *p* < 0.05). For each experimental condition, values are reported as the mean ± SD. Statistical analysis was performed via two-way ANOVA (*n* = 14). Abbreviations: HA = hydroxyapatite scaffold; SC1 = scaffold functionalized with phage clones.

The osteo-inductive ability of the two types of scaffolds (HA and SC1) was evaluated ex vivo via an immunohistochemical analysis of osterix and osteonectin at 2, 4, 8 and 16 weeks after their subcutaneous implantation into the backs of mice. Data obtained largely corroborate those observed in vitro. In particular, osterix expression (Figure 6) detected at 2 weeks in HA, progressively increased up to 8 weeks, showing a decline at the last detection time (16 weeks).

Observing data from SC1, it was found that osterix expression at 2 weeks is significantly higher than HA (up to 30% increase). Although osterix expression decreases at later timepoints, it remains consistently higher in SC1. Once again, as observed in in vitro experiments with HA scaffolds, this early osteoblast marker slowly increases in the early differentiation stage and declines at later stages. Instead, the accelerated differentiation process supported by SC1 at 2 weeks decreases in the following weeks.

**Figure 7:**
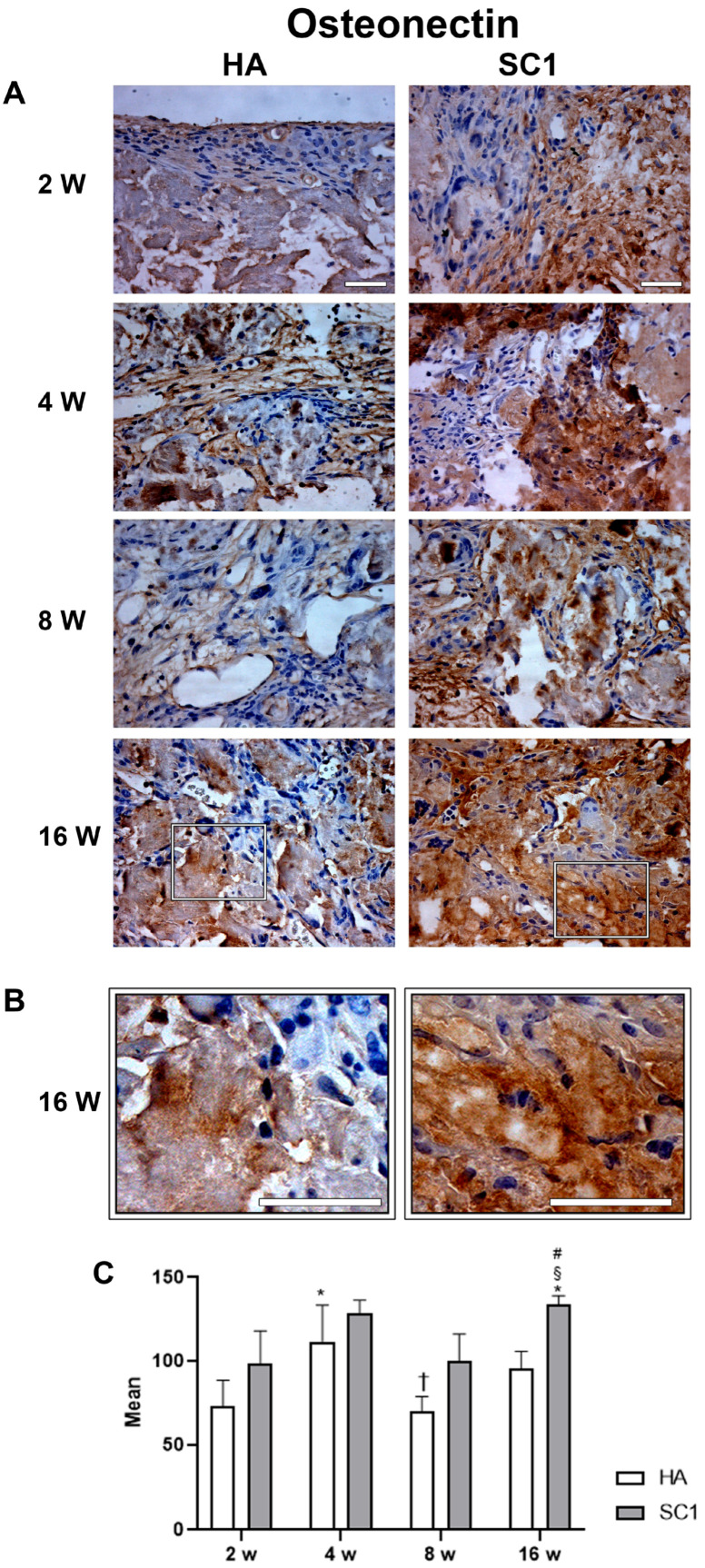
Photomicrographs showing osteonectin immunostaining of HA and SC1 scaffolds, at different time points (2, 4, 8 and 16 weeks) after subcutaneous implantation into the backs of mice (**A**). Some enlargements at 16 weeks after implantation of each scaffold are reported in panel (**B**). Both the images and the quantitative measurements (histograms in (**C**)) show that osteonectin expression showed a similar trend in both HA and SC1, being consistently more marked in SC1. The initial increase in both scaffolds between week 2 and 4 showed a reduction at week 8 and a further increase at week 16. Scale bars: 50 µm. # SC1 vs. HA at the same time point (# *p* < 0.05); * vs. 2 weeks for the same type of scaffold (* *p* < 0.05); † vs. 4 weeks for the same type of scaffold († *p* < 0.05); § vs. 8 weeks for the same type of scaffold (§ *p* < 0.05). For each experimental condition, values are reported as the mean ± SD. Statistical analysis was performed via two-way ANOVA (*n* = 14). Abbreviations: HA = hydroxyapatite scaffold; SC1 = scaffold functionalized with phage clones.

Osteonectin expression showed a similar trend in both HA and SC1, but was consistently stronger in SC1 (Figure 7). In particular, an initial increase was found in both scaffolds between weeks 2 and 4, followed by a reduction at week 8 and a further increase at week 16. However, osteonectin expression was consistently higher in SC1 (+35%, already at 2 weeks).

## 3. Discussion

Bone tissue is continuously reshaped in the process of its remodeling. Osteoclasts continuously reabsorb bone tissue, whereas osteoblasts deposit new bone tissue. An imbalance between formation and reabsorption processes may lead to osteopenia and severe osteoporosis [24]. A variety of bone disorders, such as fractures, cancer-related bone resections and cranial and craniofacial defects have recently attracted increasing attention in the field of regenerative medicine. Different biomaterials have been tested and numerous scaffolds, either of biological or synthetic origin, are able to stimulate osteogenic MSC differentiation [25,26,27,28]. In addition, MSCs can be used as an experimental model to evaluate the functional properties of these biomaterials [29].

In the present work, scaffolds made of HA were tested both in vitro and in vivo and compared to scaffolds combined with phage proteins (SC1). Sequence analysis of the selected phage-exposed peptide (QRRAGPVPP) revealed homology with the C-terminal region of SPARC (osteonectin), a key regulator of extracellular matrix mineralization and osteoblast differentiation. The alignment occurs on shared motifs (QRR, AGP, VPP) [30] between the peptide and SPARC, suggesting a mimotopic interaction. These structural similarities support the hypothesis that the peptide may contribute to the osteoinductive potential of SC1 scaffolds by functionally mimicking the SPARC domains involved in bone matrix regulation.

The initial experiments were carried out in vitro to test scaffold biocompatibility and their osteogenic induction capacity. Indeed, hematoxylin/eosin and DAPI staining at different time points confirmed that both scaffolds had excellent biocompatibility. Moreover, our results showed that the SC1, more than HA, is able per se to induce hASC osteogenic differentiation in vitro, as assessed via Alizarin Red S staining and the expression of specific markers such as osterix and osteonectin. Based on Alizarin Red S staining, our results confirmed that hASCs seeded in the scaffolds did not lose their capacity to differentiate into osteoblasts, especially when using OM. In particular, better than HA, SC1 was able to stimulate hASC osteogenic differentiation even in the absence of specific inducing factors; obviously, the presence of these factors contained in the specific medium enhances this process.

Osterix is well known to be essential for osteoblast differentiation and bone formation. It is early involved in and induces pre-osteoblasts towards immature osteoblasts [24]. Emerging evidence suggests that osterix not only plays an important role in intramembranous bone formation, but also promotes endochondral ossification by contributing to terminal cartilage differentiation. It induces the expression of several mature osteoblast genes such as collagen type-I a1, osteonectin, osteopontin, osteocalcin and bone sialoprotein, which are involved in physiological osteoblast activity during the formation of ossified bone. Osterix is also essential for embryonic skeletal development, postnatal skeletal growth and homeostasis, as well as the maturation and function of osteocytes postnatally [31]. An osterix interaction with a specific site in the sclerostin promoter suggests that it is also involved in mature osteocyte function [31]. In agreement with its physiological role, osterix starts to accumulate in the nucleus at early stages and slowly decreases towards the end of osteogenic differentiation [32].

Osteonectin, also known as SPARC or basement membrane protein 40 (BM-40), is a 32 kDa calcium-binding protein of the extracellular matrix. It represents one of the most abundant non-collagenous proteins in mineralized tissues, and it is secreted by osteoblasts during bone formation [33]. Previous studies have confirmed that osteonectin controls osteoblast mineralization through the P38 pathway [34], also influencing cell adhesion and motility. Its importance has been fully verified by numerous studies on osteonectin-null mice, where a lack of organized collagen and a low bone-formation rate were observed [35,36,37]. Moreover, it has a high affinity for type I collagen and HA, and it is expressed at high levels in bone tissue with high turnover (i.e., active osteoblasts, bone marrow progenitor cells, hypertrophic chondrocytes) [37].

Our in vitro results showed that osterix and osteonectin were expressed more in SC1 as compared to HA, also showing an earlier appearance. However, when high levels were reached at day 14, in both HA and SC1, a reduction was observed at later time points. This late reduction can be explained by taking into account that osterix is an early marker of pre-osteoblasts. Thus, the earlier the differentiation process occurs, the sooner the osterix levels decline. It is likely that the high SC1-induced differentiation stage observed here cannot be further enhanced. This interpretation is also supported by immunohistochemical data investigating osteonectin levels. In fact, whereas it gradually increases in HA from day 14 to day 46, high levels were detected in SC1 already at day 14 and maintained for the following period. Since osteonectin is considered a marker of osteoblasts at later stages of differentiation, the high expression found in SC1 already at day 14 would confirm that, in these scaffolds, an accelerated differentiation process occurs.

In in vivo experiments, animals remained healthy during the course of the study, with no evident side effects. When an ex vivo histological evaluation of the scaffolds was carried out, it was confirmed that these biomaterials were able to attract host resident cells and induce osteogenesis, thus promoting ectopic bone regeneration. This was more evident when using SC1, where the peak level of osterix immunoreactivity was observed as early as 2 weeks, whereas in HA, the peak was delayed at 8 weeks. The progressive decline of osterix immunoexpression would be explained by the physiological features of this transcription factor, which is mainly expressed at the initial phases of differentiation, decreasing as the differentiation progresses. Therefore, the use of SC1 accelerates the osteoblast differentiation of resident cells. The higher levels of osteonectin expression at all time points after implantation confirm that SC1 promotes better osteogenic differentiation compared to HA. The cyclical trend detected in both types of scaffolds might be due to its physiological turnover, since this protein is differently expressed in the course of bone remodeling [38].

Overall, our results confirm that HA scaffolds are safe biomaterials suitable for tissue engineering, and that their osteoinductive capacity can be improved through functionalization. In addition, the combination of functionalized scaffolds and mesenchymal stem cells might represent a further improvement of this strategy.

Compared to BMP-2 mimetic peptides [39], SC1 scaffolds functionalized with phage peptides offer significant advantages. Phage peptides are easy to produce at scale, inexpensive and do not induce inflammatory or immune responses [40]. Although no direct comparison has been conducted, SC1 has shown strong intrinsic osteoinductive properties even in the absence of osteogenic stimuli, supporting its translational relevance.

Given its ability to induce osteogenesis without the addition of exogenous growth factors, SC1-functionalized scaffolds could be particularly useful in pathological bone loss conditions, such as osteoporosis, where the endogenous regenerative potential is compromised.

Moreover, the use of phage-derived peptides may confer additional advantages in infectious environments, as bacteriophage components have been explored for their antibacterial properties. Future studies should investigate SC1 scaffolds in disease models of osteoporosis to evaluate both osteogenic and antimicrobial synergy, potentially opening new avenues for dual-functional scaffolds.

The in vivo model used in this study only evaluated an ectopic bone formation following the subcutaneous implant of scaffolds. Although results provide information for assessing their osteoinductive capacity, it does not fully replicate the mechanical and biological complexity of orthotopic bone repair. Future research will focus on evaluating SC1-functionalized scaffolds in models of critically sized bone defects at load-bearing sites, determining their long-term integration, mechanical performance and vascularization. Furthermore, studying the immunomodulatory effects of SC1 and its impact in pathological contexts such as osteoporosis or infectious bone loss would expand its translational potential.

The findings from this study suggest that SC1-functionalized HA scaffolds could represent a valuable clinical tool for bone regenerative medicine, especially in cases where conventional bone grafts are contraindicated or ineffective. The use of a phage-display-derived peptide mimicking osteonectin offers a biomimetic and potentially safer alternative to recombinant growth factors, avoiding the associated complications such as ectopic bone formation or tumorigenesis. Moreover, the ability of SC1 to promote osteogenesis even in the absence of osteogenic medium suggests its suitability for in situ bone regeneration.

## 4. Materials and Methods

### 4.1. Scaffold Structure and Preparation

The scaffolds (diameter of 6 mm, thickness of 4 mm) were composed of two layers made of equine type I collagen and rt in different ratios, to mimic bone composition. The first layer was made of a combination of type I collagen and MgHA in a nominal ratio of 60%/40%, while for the second layer, type I collagen and MgHA were blended in a nominal ratio of 30%/70%. The layers were synthesized separately using a standardized process, starting from an atelocollagen aqueous solution (1%, *w*/*w*) in acetic acid, by nucleating bone-like nanostructured MgHA into self-assembling collagen fibers. The self-assembled fibers were then chemically crosslinked at room temperature by BDDGE (1,4-butanediol diglycidyl ether; Merck, Darmstadt, Germany), freeze dried and gamma-sterilized at 25 kGray.

The amount of SC1 phage solution used to functionalize each scaffold was based on previous studies employing phage-display-derived peptides for biomaterial surface modification [41]. This concentration was selected to ensure a homogeneous distribution and stable conformational presentation of the peptide on the scaffold surface, while preserving biocompatibility and minimizing cytotoxicity.

### 4.2. Phage Selection, Identification, and Propagation

A 9-mer random peptide phage display library constructed using the pC89 vector with random oligonucleotides between codons 3 and 5 of the major coat protein gene M13 (pVIII), which was used for the biopanning steps [42].

To identify clones with high affinity for bone-associated targets, the library was screened on a suspension of bone marrow-derived cells [43]. Three rounds of biopanning were conducted to enrich the affinity of phages exhibiting high binding specificity. After each round, bound phages were eluted under acidic conditions, amplified in Escherichia coli TG1 and titrated. Recombinant clones were identified through blue/white screening on LB agar plates supplemented with X-Gal, IPTG and ampicillin. Blue positive colonies, indicative of phage clones harboring peptide inserts, were isolated after the third round and analyzed individually. DNA inserts from selected clones were amplified via PCR using M13-specific primers and subsequently sequenced. The amino acid sequences were aligned according to their similarity by using Clustal X 2.1 (available at [http://clustalx.software.informer.com/2.1/], accessed on 15 April 2024). GeneDoc (available at [https://genedoc.software.informer.com/amp/2.7/], accessed on 15 April 2024) was used as a tool for visualizing, editing and analyzing multiple sequence alignments of the peptides [44,45]. Amplification of the selected phage was performed in E. coli TG1 grown in LB medium containing ampicillin. Phage particles were recovered from the culture supernatant via PEG/NaCl precipitation and concentrated in TSB to a final titer of 1 × 10^13^ transduction units (TU)/mL. To remove traces of bacterial LPS, the solution was treated with 2% (*v*/*v*) Triton X-100, followed by two cycles of centrifugation at 15,000× *g* at 4 °C for 45 min. To use the phage at the final concentration of 1 × 10^11^ TU/mL, the purified phage was diluted in sterile ultrapure water, and then, the solution was filtered through 0.45 µm filters. The 6 mm Finceramica/Bone++ Osso discs were handled aseptically, and each side of the discs was soaked with 20 µL of phage solution before in vitro tests or in vivo implantation.

### 4.3. hASC Cultures

hASCs were isolated from healthy donor-derived lipoaspirates obtained after surgical procedures carried out at the Cannizzaro Hospital (Catania, Italy). Donors signed informed consent to use the lipoaspirate for experimental procedures, which were carried out in accordance with the Declaration of Helsinki and approved by the local ethics committee (Comitato etico Catania1; Authorization n. 398/202l/EMPO). The donors were three females (32–38 years old), non-smokers and occasionally taking non-steroidal anti-inflammatory drugs.

The lipoaspirate was processed as previously described [46]. Briefly, it was incubated at 37 °C with 0.075% type I collagenase (17100-17, Invitrogen, Milan, Italy) for 3 h. Following centrifugation at 1200 rpm for 10 min, pellets were resuspended in phosphate-buffered saline (PBS; Invitrogen), and the cells were filtered through a 100 μm nylon cell strainer (Falcon BD Biosciences, Milan, Italy). Finally, they were seeded in 75 cm^2^ flasks (Falcon BD Biosciences) with complete low-glucose DMEM (21885-025, Gibco, Monza, Italy) containing 10% FBS (F9665, Sigma-Aldrich, Milan, Italy), 1% penicillin/streptomycin and 1% MSC growth supplement (MSCGS; 7552, ScienCell Research Laboratories, Milan, Italy) and incubated at 37 °C with 5% CO_2_. When about 80% confluence was reached, all cultures were trypsinized, and cells were plated for expansion for three passages before the experimental procedures. As described in previous works [46], some culture samples were used to verify the stem cell nature based on the expression of positive mesenchymal (CD44, CD73, CD90 and CD105) markers and negative hematopoietic (CD14, CD34 and CD45) markers.

### 4.4. In Vivo Experiments

In vivo experiments were performed on 8 week-old BALB/c female mice (Charles River Laboratory, Boston, MA, USA), weighing 20 ± 3 g. To prevent animal suffering, mice were housed in groups of 3 or 4 per cage (IVC cages, 10.25”W × 18.75”D × 8”H), avoiding the stress associated with single housing. Animals were kept at controlled humidity and temperature (20–23 °C), throughout the entire duration of the experiment, with food and water ad libitum and a 12 h light/dark photoperiod. All procedures were carried out at the Center for Advanced Preclinical In vivo Research (CAPIR), University of Catania, and conformed to the guidelines of the Institutional Animal Care and Use Committee (I.A.C.U.C) of the University of Catania (613/2023-PR of the 29.06.2023) in accordance with the European Communities Council directive and Italian regulations (EEC Council 2010/63/EU and Italian D.Lgs. 26/2014).

### 4.5. Experimental Design

In vitro experiments were aimed at assessing the scaffold biocompatibility and osteogenic differentiation ability of seeded hASCs. In vivo experiments were aimed at evaluating the osteoinductive potential of cell-free scaffolds of each type towards mouse resident cells.

#### 4.5.1. Scaffold-Supported hASC Osteogenic Differentiation In Vitro

hASCs were used to test scaffold biocompatibility and osteogenic differentiation. For this purpose, 1 × 10^6^ hASCs were seeded dropwise on the surfaces of HA or SC1 scaffolds and incubated for 2 h at 37 °C and 5% CO_2_ before the addition of further basal growth medium. The next day, the medium was replaced with fresh DMEM or OM (BulletKit LOPT-3002, Lonza, Basel, Switzerland). Four experimental groups were then obtained: (1) HA scaffolds seeded with hASCs cultured in DMEM; (2) HA scaffolds seeded with hASCs cultured in OM; (3) SC1 scaffolds seeded with hASCs cultured in DMEM; (4) SC1 scaffolds seeded with hASCs cultured in OM. For each group, the degree of biocompatibility and of osteogenic differentiation were evaluated after 14, 28 and 46 days.

#### 4.5.2. Scaffold-Supported Osteogenic Differentiation In Vivo

A total of 56 mice were used in this study, divided into two main experimental groups, as follows: (a) Control animals (HA), in which nonfunctionalized HA scaffolds were implanted (28 mice); (b) Scaffold1 (SC1) animals, where functionalized scaffolds were implanted (28 mice,). As shown in Table 1, mice of each group were further subdivided to be monitored at four different time points (7 mice of each group for each time point).

Scaffold implantation was performed under gas anesthesia using a mixture of O_2_ and isoflurane (4% for induction, 2% for maintenance). The animals were first shaved in the dorsolateral region using an electric clipper and then sterilized with povidone–iodine. A small incision was made in the skin, creating a subcutaneous pocket for scaffold implantation. The incision was then closed using surgical sutures. Post-surgery, the animals were kept at a controlled temperature (37°) using a heating pad, until fully recovered from anesthesia. During all the experimental procedures, animal welfare was monitored weekly through observations of clinical signs (weight, movement impairments, fur appearance, consumption of food and water). At the end of each time point, the animals were sacrificed through a carbon dioxide (CO_2_), overdose and the scaffolds were explanted and processed for ex vivo histological and immunohistochemical analyses.

### 4.6. Histological Procedures

Scaffolds from in vitro or in vivo experiments were treated as previously described [47]. Briefly, at each time point and immediately after animal sacrifice, scaffolds were collected and fixed (4% paraformaldehyde) for 2 h, dehydrated, enclosed in paraffin, cut into 5–10 µm-thick sections using a microtome and placed on a polylysinated glass slides. After deparaffinization, sections underwent subsequent procedures. To evaluate scaffold biocompatibility by assessing the presence and cell density inside the scaffold, hematoxylin/eosin (Sigma-Aldrich) staining was performed as previously reported [5]. For cell counts, nuclei were stained via incubation for 10 min with DAPI. Alternate sections were also stained with Alizarin Red S (Sigma-Aldrich) to estimate the presence of calcium deposits. Alizarin Red S solution was prepared according to the manufacturer protocol: sections were incubated for 5 min, washed several times to remove the excess of the staining solution and, finally, mounted.

### 4.7. Immunohistochemical Procedures

Cell osteogenic differentiation within the scaffolds was verified based on the immunoexpression of specific markers. For this aim, two sets of sections were treated with a citrate solution, 3% H_2_O_2_ in PBS and 0.3% triton in PBS, before incubation for 1 h at room temperature with primary antibodies: anti-osterix (Rb 1:100; orb500983, BiorByt, Cambridge, UK) or anti-osteonectin (Rb 1:50; LS-C161424, LS-Bio, Newark, CA, USA). Sections were then incubated with a secondary biotinylated antibody (1:200 horse anti-mouse/rabbit, BA-1300, Vector laboratories, Newark, CA, USA) for 30 min, in an avidin/biotin complex (ABC; PK-7100, Vector laboratories) for a further 30 min and, finally, in diaminobenzidine solution (DAB; 1.02924.001, Merk, Darmstadt, Germany) for 10 min to detect peroxidase activity. The slides were then treated with hematoxylin to counterstain cell nuclei for the subsequent microscope observations.

Digital images were obtained using a Leica microscope equipped with a computer-assisted camera (Leica application suite 2.8.1, Wetzlar, Germany). For each condition, the intensity of DAB staining was measured using imageJ/Fiji (2.14.0), based on color deconvolution, to obtain pure DAB masks. The threshold for each staining was set as the average threshold values.

For in vitro experiments, the analysis was performed on two microphotographs resulting from each condition of each of the three independent experiments. Since experiments were performed in triplicate, a total of 18 microphotographs were considered for statistical analysis for each condition (*n* = 18).

For in vivo experiments, the analysis was performed on two microphotographs resulting from each of seven scaffolds implanted in mice. Thus, a total of 14 microphotographs were considered for statistical analysis for each condition (*n* = 14).

Identical thresholds for each individual staining were applied to all samples. The software measured the mean of the optical density (mean = integrated density/area).

### 4.8. Statistical Analysis

Statistical analysis was performed by using GraphPad Prism 8.0.1 (GraphPad Software, La Jolla, CA, USA). For each experimental condition, values are reported as the mean ± SD. Differences between samples were assessed using three-way analysis of variance (three-way ANOVA) followed by a post hoc Tukey’s multiple comparisons test for in vitro experiments and two-way analysis of variance (two-way ANOVA) followed by a post hoc Tukey’s multiple comparisons test for in vivo experiments. *p* values of 0.05 or less were considered statistically significant. Every possible comparison between the study groups was considered.

## Figures and Tables

**Table 1:** Experimental groups.

Study Groups	Time Points	Number of Mice
HA	2, 4, 8, 16 weeks	*n*. 28 (7 × each time point)
SC1	2, 4, 8, 16 weeks	*n*. 28 (7 × each time point)

## Data Availability

Data described in the manuscript is available upon request from the corresponding author.

## Abbreviations

The following abbreviations are used in this manuscript:

BDDGE	1,4-butanediol diglycidyl ether
DMEM	Dulbecco’s modified Eagle’s medium
FBS	fetal bovine serum
HA	hydroxyapatite
hASC	human adipose-derived mesenchymal stem cell
MgHA	Mg-substituted HA
MSC	mesenchymal stem cell
MSCGS	MSC growth supplement
OM	osteogenic medium
PBS	phosphate-buffered saline
SC1	scaffold functionalized with phage clones
TGFβ	transforming growth factor beta
TRIP-1	TGFβ receptor-interacting protein 1
